# Superior Mesenteric Artery Syndrome in a Patient with Cerebral Palsy

**DOI:** 10.1155/2014/538289

**Published:** 2014-06-26

**Authors:** Adi Neuman, Bhavita Desai, Daniel Glass, Wassim Diab

**Affiliations:** Department of Internal Medicine, Staten Island University Hospital, 475 Seaview Avenue, Staten Island, NY 10305, USA

## Abstract

Superior mesenteric artery syndrome involves compression of the third part of the duodenum due to narrowing of the area between the aorta and the superior mesenteric artery (SMA). We will describe the case of a 34-year-old with cerebral palsy who presented with abdominal pain, nausea, vomiting, and weight loss and was diagnosed with SMA syndrome via CT-imaging. With failure of conservative measures, our patient underwent a duodenojejunostomy after which improvement in her weight as well as relief of her abdominal symptoms was noted. Given the rarity of this syndrome, physicians need to keep a high index of suspicion in order to prevent the damaging consequences.

## 1. Introduction

SMA syndrome, also known as Wilkie's syndrome, is a rare cause of upper gastrointestinal obstruction associated with compression of the duodenum between the abdominal aorta and the SMA. Initial complaints include abdominal pain, nausea, vomiting, weight loss, and early satiety. Given the nonspecific nature of these symptoms, imaging is highly diagnostic in revealing the decreased aortic-mesenteric artery angle resulting in the duodenal obstruction. Initially, conservative measures including nasogastric tube decompression, correction of underlying electrolyte abnormalities, and nutritional support are sufficient to alleviate the symptoms. However, when conservative methods fail, several surgical options are available.

A misdiagnosis could lead to complications such as severe electrolyte abnormalities as well as gastric perforation from the obstruction. Mortality can be as high as 30% [1, 2] of affected cases, thereby rendering this syndrome as pertinent for physicians to understand and keep on their list of differential diagnosis.

## 2. Case History

A 34-year-old female with a history of cerebral palsy, profound mental retardation, and seizure disorder presented to the emergency department for multiple episodes of vomiting for two weeks associated with decreased oral intake. The patient was bedbound at home and required assistance for all activities of daily living. Due to her medical conditions, she was unable to provide a detailed history; however, her family reported over 10 episodes of green, bilious emesis in the 24 hours prior to her arrival. The family denied the presence of fever, diarrhea, or any sick contacts. It was unclear whether she had lost significant weight in the months prior to presentation; however, the family reported having difficulty feeding her due to progressively worsening agitation. Upon further review of systems, the patient's last seizure was a generalized tonic-clonic seizure that occurred three days prior to her presentation. The patient had no known medication allergies and was taking Lamitrogine and Zonisamide.

Upon arrival to the hospital, the patient's vital signs were stable, with a rectal temperature of 96.8 F, blood pressure of 111/66 mm of Hg, and a pulse rate of 84 beats per minute. She was breathing comfortably on room air. Height was 4′11′′ (150 cm), weight was 60 lbs. (27 kg), and BMI was 12 kg/m^2^. On physical examination, the patient appeared cachectic. She was awake and alert but unable to follow commands. The abdomen was mildly distended without evidence of tenderness or guarding. Neurological findings revealed bilateral spasticity and hyperreflexia affecting all extremities.

Laboratory tests were significant for a white cell count of 15,000 cells per cubic millimeter, sodium of 133 mmol/liter, and calcium of 10.4 mmol/liter. A urine pregnancy test was negative. Other laboratory results were within normal limits.

**Figure 1:**
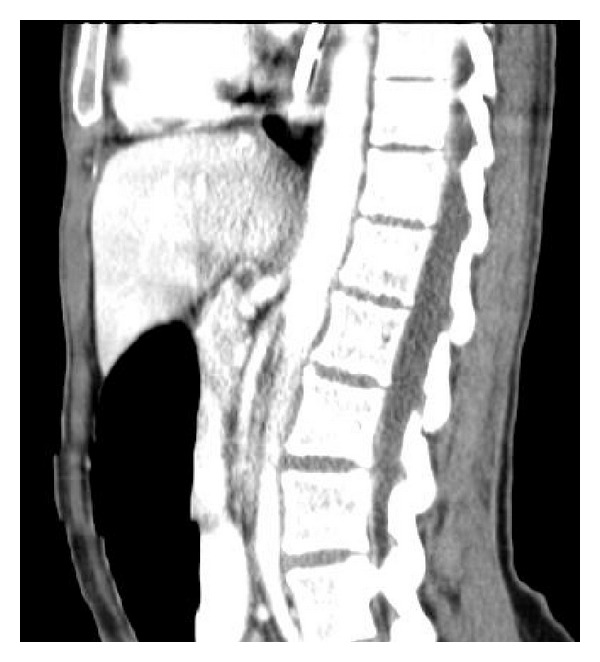
Cross-sectional imaging demonstrating the reduced aortic-mesenteric artery angle.

**Figure 2:**
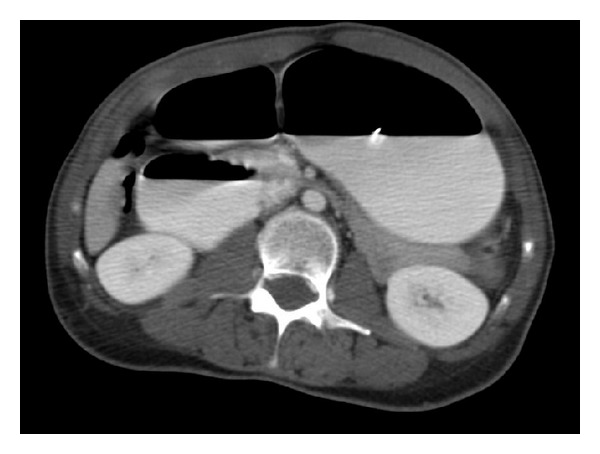
CT-imaging demonstrating SMA syndrome with resulting compression of duodenum due to aorta and superior mesenteric artery.

The patient was given Ondensetron for nausea relief, kept NPO, and given fluids intravenously. Next, CT-imaging of the abdomen and pelvis was performed with oral and intravenous contrast, which revealed distension of the stomach as well as of the first and second portions of the duodenum up to 3.6 cm, as well as cholelithiasis (Figures 1 and 2). The superior mesenteric artery was found to descend from the aorta at an angle of approximately 15°, a finding consistent with superior mesenteric artery syndrome. The patient then had an esophagogastroduodenoscopy performed on the second day of admission, which was significant for a proximal esophageal web, esophagitis, multiple ulcers in the antrum of the stomach, and a normal pylorus. No duodenal intraluminal lesions were identified. Given the finding of SMA syndrome on imaging, the decision was made to manage the patient conservatively and her diet was advanced. The patient was discharged on the fourth day of hospitalization after tolerating an oral diet.

Approximately one month following the initial admission, the patient was readmitted with similar symptoms. Since the patient had failed conservative measures for treatment of SMA syndrome, surgery was the next best option and a laparoscopic duodenojejunostomy was performed. Nearly 6 months after surgery, the patient was weighed at 68 lbs (31 kg) with a BMI of 13.7 kg/m^2^, indicating improvement in the patient's weight and nutritional status. The patient had no further reported vomiting, though she continued to have medical issues related to agitation and seizures.

## 3. Discussion 

SMA syndrome was first described by Von Rokitansky in 1861 [3]. It has an incidence of 0.013–0.3% in the general population with a mortality rate of 33% [1, 2]. The syndrome remains a rare cause of proximal intestinal obstruction occurring due to compression of the duodenum between the aorta and the SMA. Normally, the third portion of the duodenum is suspended by the ligament of Treitz in the narrow angle created between the aorta and the SMA. Any variation in the acute nature of this angle can lead to compression of the duodenum, thereby causing nausea, abdominal pain, and postprandial bilious emesis [4]. SMA syndrome can be quite extensive even leading to compression of the left renal vein and hence leading to nutcracker syndrome [3]. In the majority of patients, the normal angle between the SMA and the aorta is 45–60° using biplanar angiography versus multislice CT angiography ranging from 28 to 65°; however, in SMA syndrome, the angle can be narrowed to as low as 6° which minimizes the space between the SMA and aorta potentially leading to duodenal compression [1, 5]. The syndrome is attributed to the loss of the duodenal fat pad, which correlates with a decrease in BMI [6].

The syndrome may be due to severe rapid depletion of mesenteric fat caused by weight loss in high catabolic states such as anorexia nervosa, malabsorption, burns, and cancer [6]. It should be included in the differential diagnosis of acute onset of nausea and vomiting in individuals with those medical conditions. In addition, the syndrome has been associated with neurological injury resulting in spasticity, including traumatic brain injury and cerebral palsy, as in our patient, or following spinal surgery for scoliosis or trauma.

Diagnosis can be confirmed with imaging such as upper gastrointestinal series or computed tomography. These studies demonstrate dilation of the stomach and duodenum with an abrupt cutoff at the 3rd portion of the duodenum resulting from a reduced angle. Patients can be managed either conservatively or with surgery. Medical intervention includes nasogastric decompression and electrolyte management in the acute phase, followed by frequent, small, high-calorie feedings with changes in position such as left lateral decubitus position to bypass the obstruction. Different surgical approaches are possible for patients for whom medical intervention is unsuccessful and include lysis of the ligament of Treitz, also known as Strong's procedure, gastrojejunostomy, and duodenojejunostomy. Although initially successful, lysis of the ligament can lead to recurrence and failure of the procedure in 21% of patients. In these cases, the surgery of choice when enterotomy cannot be avoided would be laparoscopic duodenojejunostomy [7].

Our case raises several salient points about SMA syndrome. First, the patient had findings of multiple antral ulcers and esophagitis diagnosed via esophagogastroduodenoscopy. SMA syndrome has been described as presenting with symptoms of esophageal reflux and esophageal stricture, which may be precipitated by gastric hypomotility and duodenal trapping. In patients who are at high risk for SMA syndrome, an upper gastrointestinal series or computed tomography may be warranted to rule out the condition. Second, esophagogastroduodenoscopy in our patient did not show an acute narrowing of the duodenum, which was expected based on CT findings. This may have reflected the fact that duodenal obstruction in SMA syndrome is highly positional and may be partially relieved with insufflation during the esophagogastroduodenoscopy procedure.

Often patients with cerebral palsy or delayed cognition with nonspecific complaints may experience a delay in diagnosis as symptoms may be attributed to the patient's neurological dysfunction when in fact there is an actual organic cause to the disease. Unfortunately for our patient, she was unable to communicate her symptoms and returned to the hospital multiple times for the same reason before an accurate diagnosis was made. Promoting further education to physicians about SMA syndrome recognition needs to be implemented so that an early diagnosis can be made and appropriate treatment can be administered to avoid deleterious complications and sequelae. A clinical setting of weight loss or anatomical abnormality associated with nausea, vomiting, early satiety, and anorexia plus radiological evidence of gastric and early duodenal dilation should not be discounted as simply ileus but should trigger us to think about SMA syndrome.
